# Promoting population health with public-private partnerships: Where’s the evidence?

**DOI:** 10.1186/s12889-019-7765-2

**Published:** 2019-11-01

**Authors:** Lucy A. Parker, Gustavo A. Zaragoza, Ildefonso Hernández-Aguado

**Affiliations:** 1 Department of Public Health, Universidad Miguel Hernández de Elche, Alicante, Spain; 2 CIBER Epidemiology and Public Health (CIBERESP), Madrid, Spain; 3 Aplica Investigación y Traslación S. Coop. Mad, Madrid, Spain

**Keywords:** Public private partnerships, Health promotion, Governance, Competing interests

## Abstract

**Background:**

Although public-private partnerships have become common in the health sector, the evidence supporting their effectiveness is limited, and when the products or services provided by the private partner are harmful to health inherent conflicts of interest may be difficult to overcome. The objective of this study is to appraise the evidence describing process or effectiveness of public-private partnerships (PPPs) that aim to promote population health, and analyse how characteristics such as independence or competing interests influence the results of their evaluation.

**Methods:**

We carried out a systematic search of Medline and Web of Science to identify scientific reports evaluating the process or effectiveness of PPPs that aim to promote population health. Two reviewers applied inclusion criteria, extracted and evaluated study quality. We classified PPPs according to the health problem tackled, the independence of the evaluation, and the potential for competition between business interests of the private partner and health promotion activity undertaken. We classified the conclusions of the evaluation as positive (supportive/tentatively supportive) or negative (semi-critical/critical).

**Results:**

We identified 36 studies evaluating 25 PPPs. Evaluations that were favourable to the use of PPPs in health promotion were more frequently classed as “not independent” and of poor quality. On the other hand, negative evaluations were more common when the PPP involved a private partner with a high potential for competition between the health promotion activity undertaken and their financial interests. PPPs that sought to prevent non-communicable diseases were more frequently negatively evaluated compared to PPPs tackling infectious disease or other types of health problem. Almost all of the evaluations evaluated process, with only 2 papers reporting quantitative health related outcomes.

**Conclusions:**

There is still a lack of sound evidence supporting the effectiveness of public-private partnerships in health promotion, and the evidence base is skewed by non-independent evaluations. Public health actors should abstain from engaging in agreements with industries whose business interests have a high potential for competition with the health promotion activity undertaken.

## Introduction

Public-private interactions have become common in the health sector. Governments, multilateral institutions and industries are applying these arrangements to address diverse health related issues. The origins and reach of the so called public-private partnerships (PPPs) have been thoroughly described at the global level [1–3] and have been supported by many authors [4]. Goal 17 of the United Nations Sustainable Development Goals for 2030 actively advocates for countries to “Encourage and promote effective public, public-private and civil society partnerships, building on the experience and resourcing strategies of partnerships” [5]. Arguments in favour of PPPs include that the immense threats to health cannot be tackled by governments alone [6]; that PPPs enrich the capacity, quality and reach of public health services [7]; that partnerships help to put health in all policies [8]; that they improve self-regulation [9]; and finally, that PPPs promote sustainable business models that allow innovation in more healthful design and content of products [10].

However, criticism has also arisen regarding PPPs in the health sector. Authors argue that alliances between public health and private sector have inherent conflicts of interest that cannot be reconciled when the products or services provided by the private partner are harmful to health [11, 12]. Collaboration in health promotion confers legitimacy and credibility on industries that produce such disease-related products and can damage the credibility of public health institutions [13]. Furthermore, public-private interactions may lead to institutional capture when companies succeed in influencing governments and multilateral institutions to undermine regulatory measures to protect population health, such as taxation [12, 14].

While many public-private interactions are used in developing, financing and providing public health infrastructure and service delivery or in providing drugs, vaccines or other products, significant criticism has arisen when it comes to agreements between governments and industries that are negatively associated with health, especially in the area of health promotion [4]. There is an important difference between health promotion and service provision in that there is limited commercial interest in health promotion initiatives. Health promotion is understood as the process of enabling people to increase control over and improve their health, covering a wide range of social and environmental initiatives to improve the adoption of healthy public policies, increase health literacy and make changes in the physical environment that ultimately make adopting healthy behaviours easier for the population. A proportion of these activities may enter in direct conflict with business models.

Some authors call for precaution when entering in partnerships for health promotion with companies that market products negatively affecting health, at least while there is no evidence of their effectiveness. Moodie R et al. suggest there is no evidence that the partnership of alcohol and ultra-processed food and drink industries is safe or effective, unless driven by the threat of government regulation [15]. Galea and McKee have recommended five tests before policy-makers engage in public–private partnerships [16]. The premise is to avoid partnerships when the core product or service provided by the company is health damaging, and to ensure their role in the partnership is to aid implementation of activities and not to develop general strategy lines, which is ultimately the responsibility of public partner alone. Recognition of the potentially damaging effects of collaboration with large corporations has grown in recent years, and the Global Fund’s decision to enter into partnership with Heineken in African countries was swiftly reversed after uproar and pressure from the civil society [17–20]. Yet, the debate continues, and earlier this year, Iliff and Jha [18] argued that limiting partnerships to companies whose products have no harms would eliminate nearly all potential opportunities for collaboration, and may seriously limit progress towards achieving global health goals. Instead they suggest that global health community evaluate all potential partnerships with more clearly enunciated, established criteria.

Beyond the need to establish criteria for engaging with private partners to implement health promotion programs, scientific evidence on the effectiveness of these partnerships becomes the key issue. Ideally evaluations should address whether or not the partnership is able to produce a real and durable improvement in population health. While it may be challenging to demonstrate changes using health indicators, intermediate proxy indicators established prior to carrying out the evaluation can be useful to indicate that the initiative is having the desired effect. The difficulty to evaluate public-private interactions, and the frequency of potential conflicts of interest could explain the scarcity of evaluations, particularly in health promotion [4]. So far, several evaluations have addressed public-private agreements on health services provision or have been restricted to evaluating features of the PPP related to successful functioning (process evaluations) rather than impact on health [21, 22]. The aim here is to identify scientific papers reporting evaluations of PPPs in health promotion in order to assess the evidence in favour of PPPs, and analyse how characteristics of the evaluations such as independence or conflicting interests may influence their results.

## Methods

### Search and selection of studies

We carried out a systematic search of the Medline database using Pubmed and Web of Science (WoS) on 27th June 2018 combining the text words “public private partnerships” OR “public private partnership” to identify studies that evaluated PPPs in health promotion. Two reviewers independently screened the titles and abstracts of all potential articles and reviewed the full text of preselected abstract for final inclusion in the study. We also reviewed the references of included papers to identify potentially relevant studies.

**Fig. 1:**
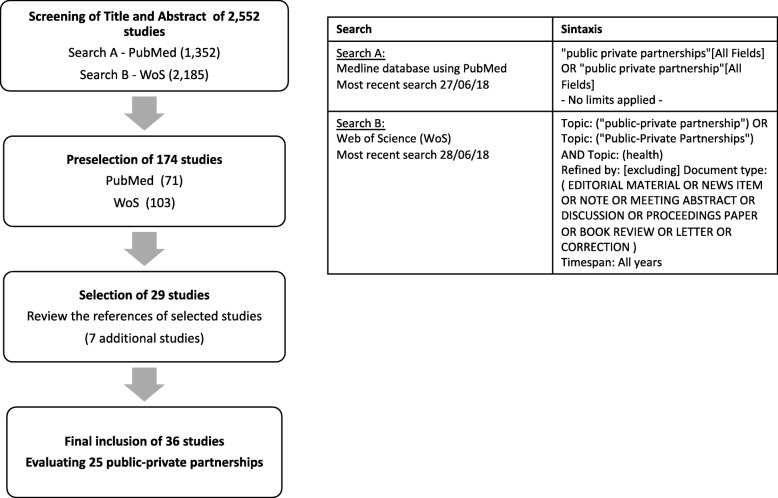
Search and selection of scientific articles that evaluate the role of a local, national or multi-national public-private partnership in health promotion

We selected studies based on the following criteria: Scientific articles published in a peer-reviewed journal that evaluated the role of a local, national or multi-national Public-Private Partnership in health promotion, understood as the process of enabling people to increase control over and improve their health. We did not include initiatives that focused solely on the provision of health care such as pharmaceuticals, vaccinations, nutritional supplements, or provision of clinical services. Furthermore, we chose to exclude initiatives where the private partner was the tobacco industry as it is already widely accepted that undue influences from the tobacco industry prevent them from having a legitimate role in health promotion. PPPs were only eligible for inclusion if they included at least one public partner and one private partner, defined as for profit companies or organizations, or foundations funded by private companies although not necessarily for profit. Initiatives were considered to be PPPs when the interaction between private and public partners was established for the specific purpose of implementing or driving forwards a health promotion program with a shared goal, thus excluding other types of interaction such as dialogue preceding regulation. Quantitative and qualitative evaluations were included if they empirically analysed either the process and/or effectiveness of PPPs in health promotion. We resolved discrepancies in the screening of abstracts by arbitration with a third reviewer. Figure 1 shows a flow diagram detailing the search and selection process.

### Data extraction

Two researchers independently extracted the characteristics of each evaluation from the articles. We classified each study as quantitative, qualitative or mixed; and whether it addressed process or impact. Furthermore, we classified each article according to the independence of the evaluation. Evaluations were considered to be independent only if all author affiliations and funding sources were unrelated to the partners involved in the PPP. Government workers were considered independent as long as they did not work directly in the area of the PPP, and authors working in publicly funded evaluations units were considered unbiased, thus independent. We categorised the private partner(s) of the PPP according to whether the business interests were likely to be in conflict with the health promotion activity undertaken by the PPP. This question was not always straightforward. For example, while fresh fruit and vegetable producers have no conflict with health promotion activities focussing on healthy weight, and junk food restaurants have a clear conflict, other types of food producers such as the meat and dairy industry, large supermarket chains or unspecified restaurants are more difficult to classify. For this reason, we classified the PPPs according to three categories (Table 1).

**Table 1:** Competition between business interests of the private partner and the health promotion activity undertaken by the PPP

Classification given	Description
None	Private partner or partners are unrelated to the health promotion activity and their products are not related negatively to health. Private partner or partners produce items with a positive impact on health (fresh fruit and vegetable producers, toothpaste, pharmaceuticals)
Some potential for competing interests	Private partner or partners produce or sells items which may have a potentially negative impact on health, especially if consumed in excess (meat industry, dairy, supermarkets.) Multiple partners involved but not enough detail provided in order to classify the PPP in the other two categories.
High potential for competing interests	Private partner or partners directly related to the health promotion activity, and at least one produces items with an established negative impact on health (alcohol, fast foods, sugary-drinks etc.)

Finally, we recorded word for word the conclusion of the evaluation according to the abstract or discussion of the paper, and classified it as critical, semi-critical, tentatively supportive or supportive.

### Evaluation of study quality

We developed a simple tool to gage study quality which incorporated dimensions from existing guidelines [23–25]. We judged this the best approach because the evaluation studies were heterogeneous using different designs and methodologies. We piloted the tool on three papers and met to discuss and make improvements/clarifications to aid application. A copy of the tool can be found in Additional file 1: Text S1. We applied the tool in duplicate.

### Data analysis

When there were numerous studies on the same PPP, we combined the findings of the evaluations for analysis. In the case of the UK Public Health Responsibility Deal, which entails voluntary partnerships with industry in four dimensions (food, physical activity, alcohol and work), we classed each dimension as a separate PPP, thereby combining the evidence provided of evaluations only when they evaluated the same dimension. The Australian Food and Health Dialogue, which later changed its name to the Healthy Food Partnership in 2015, was classed as one PPP. If the combined evaluations were from different years, we noted the year of the most recent evaluation. If there was a discrepancy among key variables, including the grading of the conclusions of the evaluation (as supportive, critical etc.), we used the information from the paper with highest quality. When quality was equal, we kept the conclusions of the most independent evaluation. We carried out a univariate analysis to assess the relationship between the conclusions of the evaluation (bivariate categorisation supportive or tentatively supportive compared to critical or semi-critical) and key characteristics of the PPP or of the evaluation. We assessed statistical association using Fisher’s exact test.

## Results

Of the thirty-six studies analysed, eleven (30.6%) were evaluating PPPs in the United Kingdom ((UK), of which nine evaluated the Public Health Responsibility Deal). Nine (25%) evaluated PPPs in the United States of America (USA), 5(13.9%) in Australia (all evaluating the Public Health Dialogue and latter Healthy Food Partnership), and 3(8.3%) in Tanzania (evaluating the National Voucher Scheme for mosquito-net distribution, or its precursor). The remaining studies were carried out in Argentina, Botswana, Canada, Malaysia, Nigeria, Sri Lanka, Zambia and one study included multiple partnerships from various countries. Less than half of the papers were deemed to be of high quality providing strong evidence (17 studies, 47.2%), while 12 studies (33.3%) were moderate quality and 7(19.4%) were poor quality. Almost all of the papers were published on or after 2012 (34 studies, 94.4%). The earliest evaluation was published in 1999, and was the KINET (a social marketing programme of treated nets and net treatment for malaria control in Tanzania) [26].

Thirty-six papers analysed 25 different public-private partnerships (Additional file 2: Text S2, List of references of selected studies; Additional file 3: Table S1, Characteristics of the PPPs evaluated). The majority of the papers assessed the effectiveness of PPP in the control and prevention of Non-Communicable Diseases (NCDs) (15 PPPs evaluated, 60%). The PPPs tended to foment action on known NCD risk factors by creating a healthier food environment (8 PPPs of which 2 sought specifically to reduce salt consumption), increasing physical activity (2 PPPs), reducing alcohol consumption (1 PPP), or a mix of these initiatives (4 PPPs). There were 7 evaluations of PPPs to tackle HIV, TB or Malaria using a health promotion approach. The remaining 3 evaluations were of PPPs tackling urban food insecurity, vaccine preventable disease, and promoting improved working conditions. Less than half of the PPPs were evaluated by a team of researchers independent from the partners involved in the partnership (10 evaluations, 40%, Table 2). More than half of the alliances involved partnership with a private partner with some potential for competition between the financial interests of the firm and the health promotion activity undertaken (10, 40.0% with high potential and 4, 16.0% with moderate potential). Thirteen of the PPPs were assessed using qualitative methods (52%). Only 2 of the evaluations included an evaluation of impact using quantifiable health-related outcomes (neither of which was judged to be a high-quality evaluation). The complete quality analysis of the 36 papers included in the study can be found in Additional file 4: Table S2.

**Table 2:** Conclusions regarding the public-private partnerships (PPP) in health promotion according to characteristics of the PPP or the evaluation

Characteristics of the PPP or the evaluation	N total^a^	Conclusions regarding PPP in health promotion		*P* Value
		Critical/Semi-critical N (%)	Supportive/Tentatively supportive N (%)	
Health problem targeted				0.040
Non-communicable Disease	15	8 (53.3)	7 (46.7)	
Infectious disease (TB, Malaria, HIV)	7	0 (0.0)	7 (100.0)	
Other ^b^	3	1 (33.3)	2 (66.7)	
Potential for conflict between business interests of private partner and the health promotion activity				0.010
High potential	10	7 (70.0)	3 (30.0)	
Moderate potential	4	1 (25.0)	3 (75.0)	
Low potential	11	1 (9.1)	10 (90.9)	
Independence of evaluation				0.000
Yes	10	8 (80.0)	2 (20.0)	
No	14	0 (0.0)	14 (100.0)	
Unclear	1	1 (100.0)	0 (0.0)	
Quality of evaluation				0.003
Strong	9	7 (77.8)	2 (22.2)	
Moderate	9	2 (22.2)	7 (77.8)	
Weak	7	0 (0.0)	7 (100.0)	
Research Type				0.412
Quantitative	7	6 (85.7)	1 (14.3)	
Qualitative	13	7 (53.8)	6 (46.1)	
Mixed-methods	5	3 (60.0)	2 (40.0)	
Evaluation Type				0.373
Impact with quantifiable health-related outcomes	2	0 (0.0)	2 (100.0)	
Process with quantifiable intermediate outcomes	11	3 (27.3)	8 (72.7)	
Process without quantifiable intermediate outcomes	12	6 (50.0)	6 (50.0)	
Total	25	9 (36.0)	16 (64.0)	

^a^Evaluations of 25 PPPs from 36 scientific articles. ^b^Urban food insecurity, health at work, vaccine preventable disease

Sixteen evaluations (64.0%) supported the application in health promotion of the PPP evaluated. Evaluations were more frequently critical if the PPP sought to prevent non-communicable diseases (*p* = 0.04, Table 2). All of the evaluations of PPPs undertaking health promotion in the area of HIV, TB or Malaria prevention had positive conclusion about the approach. It is worth remarking that none of the evaluations were independent, and none of them had private partners with business interests that were likely to be in competition with the health promotion activity undertaken (Additional file 5: Table S3). Overall, a high potential for competition between the financial interests of the private partner and the health promotion activity appeared to be significantly associated with negative conclusions regarding PPPs in health promotion (7 evaluations, 70%, *p* = 0.010, Table 2). Furthermore, evaluations that supported PPPs in health promotion were more frequently classed as “not independent” (*p* < 0.001) and deemed to be of poor quality (*p* = 0.003, Table 2). A significant proportion of the process evaluations were critical of the PPPs, particularly those that were purely qualitative.

## Discussion

In spite of recent efforts to evaluate several well-known public-private interactions, there is still a lack of sound evaluation of PPPs in terms of health results. Most of existing evaluations focused on the process of the public-private arrangements. While partnerships to address infectious diseases are more frequently reported as successful, this is likely to be related to the fact that health promotion activities related to infectious disease will rarely be in conflict with business interests of the private partner (and any association likely to be a positive e.g. healthier workforce, selling more mosquito nets). Conversely, for PPPs tackling NCDs, a significant proportion of the evaluations were critical of partnership with private sectors for health promotion. Positive evaluations were rarely observed in independent evaluations, and the majority of partnerships with industries that have commercial interests competing with the health promotion activity, did not support PPPs in this area.

These findings are important because NCDs continue to rise globally, particularly in low and middle-income countries (LMIC) where globalization has increased availability of tobacco, alcohol and unhealthy foods, and industry activities tend to be poorly regulated [15]. These same countries may have seen some success in public-private alliances for improving health service provision or improving research and development on drugs and vaccines for diseases disproportionately affecting the poor, although the academic literature on the subject remains limited [27]. However, these health arenas differ significantly from health promotion, which seeks to increase peoples control over and improve their health. True health promotion activities seek to change the social and environmental determinants of health to facilitate population health improvements, while conversely, industries directly related to unhealthful products push to frame the issue as a need for individual behaviour change [28]. In a political environment with limited resources addressed to health, and the need to tackle NCDs beginning to take its place in the health agendas of many LMIC, PPPs may appear to be a promising area to explore. A clear recommendation from WHO is needed to prevent governments from forming useless partnerships with industries that have competing interests, that only serve to delay legislation protecting population health, while at the same time providing the industries with a false credibility via corporate social responsibility claims.

Our results show that there is limited evidence proving the effectiveness of PPPs for health promotion, at least in the area of NCDs, and supports the idea that governments should air on the side of caution. Applying this precautionary principle could avoid confusion considering that goal 17 of the United Nations Sustainable Development Goals for 2030 actively advocates for countries to “Encourage and promote effective public, public-private and civil society partnerships, building on the experience and resourcing strategies of partnerships” [5]. Effective partnerships require the application of the principles proposed by Galea and McKee [16]. Instead of getting involved in interactions with transnational corporations that produce unhealthy products, governments could pay attention to the possibilities of engaging with business whose products are health promoting, or unrelated to health. Our results suggest that only when there is no conflict between the business interests of the private partner and the health promotion activity undertaken by the PPP are there possibilities of success. On the other hand, the potential harms to public credibility and good governance of some public-private interactions must not be forgotten [4].

The proportion of non-independent evaluations was high, and non-independent evaluations more frequently described success of the PPP under evaluation. For example, one paper that explicitly sought to document the effectiveness of PPPs in the area of childhood obesity was funded entirely by the Mondaléz foundation, which includes numerous chocolate and confectionary producers and which also made a payment directly to one of the senior authors for writing the paper [29]. We also classed evaluations written by individuals involved with the public partner as non-independent under the assumption that authors who had invested time and resources in the PPP and would have interest in demonstrating the positive impact. Although the latter may be less alarming, it all adds up to the same thing, to skew the evidence-base in favour of PPPs, and can lead to unwarranted support for this approach.

### Study limitations

We limited the review to material published in scientific peer-reviewed journals, and there are likely to be other relevant documents in grey literature (e.g. reports from multilateral organizations or non-governmental organizations) that evaluate PPPs but they were not included here. Furthermore, there may be numerous initiatives that have been evaluated ad hoc and are never formally reported. We limited our review to peer-reviewed scientific journals assuming these are more likely to be independent, methodologically sound evaluations. Nevertheless, a significant proportion of the studies evaluated were methodologically poor and more than half were written by people related to one of the partners involved in the PPP. Internally-evaluated initiatives, publishing detailed and critical evaluations of the implementation process or monitoring of public-private partnerships may be useful for making operational improvements to new or existing initiatives, but it is clear that a partner who has dedicated time and resources to implementation, is likely to have an interest in demonstrating their worth. Although they can be useful for some endeavours, they cannot substitute the requirement for un-biased independent evaluations that measure indicators related to the effectiveness of the health promotion activity undertaken as it was initially set out.

The evaluation of study quality was challenging because the studies used different designs and methodologies. We developed a simple tool that incorporated dimensions from existing guidelines [23–25], but must recognise that using a new tool that has not been previously validated may limit the validity of our composite measure of study quality.

Regarding the search strategy, we systematically searched two databases using the search term “public private partnerships” OR “public private partnership”. Although it is possible that authors of relevant evaluations may have used different terms (such as intersectoral collaboration, alliance, or coalition), after numerous pilot searches with different terms prior to choosing this strategy, and after reviewing the references of all included articles, we deemed the strategy used to be sufficiently sensitive to identify a large proportion of relevant evaluations. We acknowledge that some papers evaluating PPPs as defined here are likely to have been missed because they used different terminology. This limitation is unlikely to introduce significant selection bias into the study because the terminology used by the authors of evaluations is not likely to be related to their independence, or other characteristics analysed. Therefore, it is unlikely to detract from the main message of the paper related to the lack of independent, unbiased evaluations of the effectiveness of PPPs for health promotion.

Lastly, inclusion of grey-literature or unpublished evaluations, would likely increase the proportion of non-independent, poor quality studies but is unlikely to change the conclusion, that there is a lack of sound evidence showing health improvements due to government partnerships with industry for health promotion.

## Conclusions

In conclusion, there is a lot of activity around the PPPs, and they appear to be widely accepted in spite of the lack of sound evidence [4, 30]. Even studies that were critical of the PPPs they evaluated, presented the issue as a need to improve their functioning and effectiveness, using the lessons learned from their evaluation rather than to avoid them completely [31, 32]. Our results back the position of ending any interaction with the food and drinks industry, at least in the field of NCDs. Before engaging in partnership with private partners, governments should apply the principle of precaution. Public health actors should abstain from engaging in agreements with industries whose business interests have a high potential for competition with the health promotion activity undertaken. Health promotion activities related to NCD prevention are particularly vulnerable to this this condition, and we found no evidence supporting their effectiveness.

## Supplementary information

**Additional file 1: Text S1.** Tool to evaluate the quality of the studies included in the review.

**Additional file 2: Text S2.** List of 36 studies evaluating PPPs included in the review.

**Additional file 3: Table S1.** Details and classification of the 25 PPPs evaluated.

**Additional file 4: Table S2.** Analysis of quality of the 36 studies evaluating PPPs included in the review.

**Additional file 5: Table S3.** Conclusions regarding the public-private partnerships (PPP) for health promotion according to characteristics of the PPP or the evaluation, stratified by health problem.

## Data Availability

All data generated or analysed during this study are included in this published article and its supplementary information files.

## Abbreviations

LMIC: Low- and Middle-Income Countries
NCD: Non-Communicable Diseases
PPP: Public-Private Partnership
