# Supplementary material for: Promoting population health with public-private partnerships: Where’s the evidence?
Source: BMC Public Health. 2019 Nov 1;19:1438. doi: 10.1186/s12889-019-7765-2 (PMC6824113; doi:10.1186/s12889-019-7765-2)
Supplement: Supplementary file 1 — Additional file 1: Text S1. Tool to evaluate the quality of the studies included in the review. [file 12889_2019_7765_MOESM1_ESM.docx]

**Text S1:** Tool for assessing risk of bias of individual studies evaluating Public-Private Partnerships in health promotion.

## **INDEPENDENCE**

### Did an independent group not related to the partnership do the evaluation of the Public Private Partnership?

- YES (author affiliation and funding source is not related to any of the partners involved in the PPP) 🡪 LOW
- UNCLEAR (it is unclear whether there is a relationship between the authors’ affiliation or funding source and the PPP. Internet search does not clarify the issue) 🡪 MODERATE
- NO (the authors of the evaluation are related to the partnership or the evaluation is financed directly by one of the partners with clear interest in the outcome) 🡪 HIGH

| **Risk of bias**  *(circle as appropriate)* | HIGH | MODERATE | LOW |
| --- | --- | --- | --- |

## **OBJECTIVES**

### Were the objectives of the evaluation clearly stated, and the outcome indicators clearly defined, in order to assess the degree of success of the partnership?

- YES (the paper provides a statement about the objective of the evaluation in terms of process or outcomes; and the results of the evaluation are reported together with a motivated explanation that lead the authors to value it as positive or negative) 🡪 LOW
- UNCLEAR (the objectives of the evaluation are not clearly stated or the procedures to assess the results of the evaluation are not clearly reported) 🡪 MODERATE
- NO (there is a lack of information about objectives and ways to evaluate the effects of the partnership) 🡪 HIGH

| **Risk of bias**  *(circle as appropriate)* | HIGH | MODERATE | LOW |
| --- | --- | --- | --- |

## **MEASURMENT-DESIGN**

### A) Is the evaluation analytical in that it compares data over time (before-after, time-series) or between populations (controlled trials)? and if so, are the procedures for data collection the same for all time points or populations being compared?

- YES (the procedures for data analysis are the same for all time points or populations) 🡪 LOW
- UNCLEAR (if it is not clear in the paper, e.g. dates of collection are not mentioned in the text) 🡪 MODERATE
- NO (no comparison made, the study only presents data at one time point or in a single intervention population. Or the procedures for data collection are not the same) 🡪 HIGH
- N/A (this is study analysing process, comparative analysis irrelevant)

| **Risk of bias**  *(circle as appropriate)* | HIGH | MODERATE | LOW |
| --- | --- | --- | --- |

B) Is it likely that the observed changes in the outcomes are due to the PPP and independent of other changes?

- YES (there were no other changes that affect outcomes over the time of intervention or the authors give a rationale to assume the unlikelihood that the observed outcomes have other cause) 🡪 LOW
- UNCLEAR (there is incomplete information to assure independence) 🡪 MODERATE
- NO the changes in outcome could have other explanations. 🡪 HIGH
- N/A (this is study analysing process, comparative analysis irrelevant)

| **Risk of bias**  *(circle as appropriate)* | HIGH | MODERATE | LOW |
| --- | --- | --- | --- |

## **OUTCOME MEASUREMENT** (process indicators or health outcomes)

A) Quantitative measurements: Were the measurements of the primary outcomes reliable?

- YES (the origin of data –documents, population rates, etc.- are described in detail; are reliable –e.g. official surveillance, government publications-; and unlikely to be susceptible to observer bias ) 🡪 LOW
- UNCLEAR (there is insufficient information to judge reliability of outcome data) 🡪 MODERATE
- NO (the outcomes were measured with high risk of bias (e.g. incomplete records used and likely to be susceptible to selection bias) 🡪 HIGH
- N/A

| **Risk of bias**  *(circle as appropriate)* | HIGH | MODERATE | LOW |
| --- | --- | --- | --- |

B) Qualitative evaluations: Were the measurements of the primary outcomes reliable?

- YES (there is enough information about types of data collected; details of data collection procedures and description of instruments; and, description of techniques to enhance trustworthiness – e.g. member checking, audit trail, triangulation-. The authors also discuss the method applied and how their choice influence study conclusions) 🡪 LOW
- UNCLEAR (the description of the methodology to assess the outcomes of the PPP are incomplete) 🡪 MODERATE
- NO (the study is highly susceptible of measurement bias, data gathered through interviews to individuals relevant to the partnership interested in the outcome of evaluation) 🡪 HIGH
- N/A

| **Risk of bias**  *(circle as appropriate)* | HIGH | MODERATE | LOW |
| --- | --- | --- | --- |

## **GLOBAL RATING**

Global rating for the evidence supplied by this paper

- Strong (no high risk of bias ratings, and at least one low risk of bias rating)
- Moderate (one high risk of bias rating; or two or more moderate risk of bias)
- Weak (two or more high risk of bias ratings)

Are there any discrepancies between the two reviewers with respect to component ratings? YES / NO

If yes, how many:

If yes, indicate the reason for discrepancy:

- 1. Oversight
  2. Differences in interpretation criteria
  3. Differences in interpretation of the study

Final decision of both reviewers: 1 Strong

2 Moderate

3. Weak
