# Supplementary material for: Promoting population health with public-private partnerships: Where’s the evidence?
Source: BMC Public Health. 2019 Nov 1;19:1438. doi: 10.1186/s12889-019-7765-2 (PMC6824113; doi:10.1186/s12889-019-7765-2)
Supplement: Supplementary file 2 — Additional file 2: Text S2. List of 36 studies evaluating PPPs included in the review. [file 12889_2019_7765_MOESM2_ESM.docx]

**Text S2:** List of Articles included in the Review.

1. Gorham G, Dulin-Keita A, Risica PM, Mello J, Papandonatos G, Nunn A, et al. Effectiveness of Fresh to You, a Discount Fresh Fruit and Vegetable Market in Low-Income Neighborhoods, on Children’s Fruit and Vegetable Consumption, Rhode Island, 2010-2011. Prev Chronic Dis [Internet]. 2015;12:E176. Available from: http://www.pubmedcentral.nih.gov/articlerender.fcgi?artid=4611858&tool=pmcentrez&rendertype=abstract

2. Ng SW, Popkin BM. The Healthy Weight Commitment Foundation pledge: calories purchased by U.S. households with children, 2000-2012. Am J Prev Med [Internet]. 2014 Oct;47(4):520–30. Available from: http://www.ncbi.nlm.nih.gov/pubmed/25240968

3. Ng SW, Slining MM, Popkin BM. The Healthy Weight Commitment Foundation pledge: calories sold from U.S. consumer packaged goods, 2007-2012. Am J Prev Med [Internet]. 2014 Oct;47(4):508–19. Available from: http://www.ncbi.nlm.nih.gov/pubmed/25240967

4. Pettygrove M, Ghose R. From “Rust Belt” to “Fresh Coast”: Remaking the City through Food Justice and Urban Agriculture. Ann Am Assoc Geogr [Internet]. 2018 Mar 4;108(2):591–603. Available from: https://www.tandfonline.com/doi/full/10.1080/24694452.2017.1402672

5. Schellenberg JR, Abdulla S, Minja H, Nathan R, Mukasa O, Marchant T, et al. KINET: a social marketing programme of treated nets and net treatment for malaria control in Tanzania, with evaluation of child health and long-term survival. Trans R Soc Trop Med Hyg [Internet]. 1999;93(3):225–31. Available from: http://www.ncbi.nlm.nih.gov/pubmed/10492745

6. Harris DM, Seymour J, Grummer-Strawn L, Cooper A, Collins B, DiSogra L, et al. Let’s Move Salad Bars to Schools: A Public–Private Partnership To Increase Student Fruit and Vegetable Consumption. Child Obes [Internet]. 2012;8(4):294–7. Available from: http://online.liebertpub.com/doi/10.1089/chi.2012.0094

7. Varda DM, Williams M V, Schooley M, Duplantier D, Newman K, Lowe Beasley K, et al. An Innovative Network Approach to Coordinating a National Effort to Improve Cardiovascular Health: The Case of Million Hearts. J Public Health Manag Pract [Internet]. 2018 Jun 7; Available from: http://www.ncbi.nlm.nih.gov/pubmed/29889170

8. Sedlmayr R, Fink G, Miller JM, Earle D, Steketee RW. Health impact and cost-effectiveness of a private sector bed net distribution: experimental evidence from Zambia. Malar J [Internet]. 2013;12:102. Available from: http://www.ncbi.nlm.nih.gov/pubmed/23506170

9. Sanders KC, Rundi C, Jelip J, Rashman Y, Smith Gueye C, Gosling RD. Eliminating malaria in Malaysia: the role of partnerships between the public and commercial sectors in Sabah. Malar J [Internet]. 2014 Jan 21;13:24. Available from: http://www.ncbi.nlm.nih.gov/pubmed/24443824

10. Asuquo AE, Pokam BDT, Ibeneme E, Ekpereonne E, Obot V, Asuquo PN. A public-private partnership to reduce tuberculosis burden in Akwa Ibom State, Nigeria. Int J mycobacteriology [Internet]. 2015 Jun;4(2):143–50. Available from: http://www.ncbi.nlm.nih.gov/pubmed/26972883

11. Shankar B, Brambila-Macias J, Traill B, Mazzocchi M, Capacci S. An evaluation of the UK Food Standards Agency’s salt campaign. Health Econ [Internet]. 2013 Feb;22(2):243–50. Available from: http://www.ncbi.nlm.nih.gov/pubmed/22223605

12. Fernando D, Wijeyaratne P, Wickremasinghe R, Abeyasinghe RR, Galappaththy GNL, Wickremasinghe R, et al. Use of a public-private partnership in malaria elimination efforts in Sri Lanka; a case study. BMC Health Serv Res [Internet]. 2018;18(1):202. Available from: http://www.ncbi.nlm.nih.gov/pubmed/29566691

13. Jane B, Gibson K. Corporate sponsorship of physical activity promotion programmes: part of the solution or part of the problem? J Public Health (Oxf) [Internet]. 2018 Jun 1;40(2):279–88. Available from: http://www.ncbi.nlm.nih.gov/pubmed/28591792

14. Leon KS, Ken I. Food Fraud and the Partnership for a ‘Healthier’ America: A Case Study in State-Corporate Crime. Crit Criminol [Internet]. 2017 Sep 15;25(3):393–410. Available from: http://link.springer.com/10.1007/s10612-017-9363-x

15. Eze IC, Kramer K, Msengwa A, Mandike R, Lengeler C. Mass distribution of free insecticide-treated nets do not interfere with continuous net distribution in Tanzania. Malar J [Internet]. 2014;13(1):196. Available from: http://www.scopus.com/inward/record.url?eid=2-s2.0-84902259876&partnerID=tZOtx3y1

16. Kramer K, Mandike R, Nathan R, Mohamed A, Lynch M, Brown N, et al. Effectiveness and equity of the Tanzania National Voucher Scheme for mosquito nets over 10 years of implementation. Malar J [Internet]. 2017;16(1):255. Available from: http://www.ncbi.nlm.nih.gov/pubmed/28619076

17. Ramiah I, Reich MR. Building effective public-private partnerships: Experiences and lessons from the African Comprehensive HIV/AIDS Partnerships (ACHAP). Soc Sci Med. 2006;63(2):397–408.

18. Elliott T, Trevena H, Sacks G, Dunford E, Martin J, Webster J, et al. A systematic interim assessment of the Australian Government’s food and health dialogue. Med J Aust. 2014;200(2):92–5.

19. Lindberg R, Nichols T, Yam C. The Healthy Eating Agenda in Australia. _Is_ Salt a Priority for Manufacturers? Nutrients [Internet]. 2017 Aug 15;9(8):881. Available from: http://www.ncbi.nlm.nih.gov/pubmed/28809812

20. Trevena H, Dunford E, Neal B, Webster J. The Australian Food and Health Dialogue - the implications of the sodium recommendation for pasta sauces. Public Health Nutr [Internet]. 2014 Jul;17(7):1647–53. Available from: http://www.ncbi.nlm.nih.gov/pubmed/23830096

21. Jones A, Magnusson R, Swinburn B, Webster J, Wood A, Sacks G, et al. Designing a Healthy Food Partnership: lessons from the Australian Food and Health Dialogue. BMC Public Health [Internet]. 2016;16:651. Available from: http://www.ncbi.nlm.nih.gov/pubmed/27465746

22. Trevena H, Neal B, Dunford E, Wu JHY. An evaluation of the effects of the Australian Food and Health Dialogue targets on the sodium content of bread, breakfast cereals and processed meats. Nutrients [Internet]. 2014 Sep 19;6(9):3802–17. Available from: http://www.ncbi.nlm.nih.gov/pubmed/25244369

23. Castronuovo L, Allemandi L, Tiscornia V, Champagne B, Campbell N, Schoj V. Analysis of a voluntary initiative to reduce sodium in processed and ultra-processed food products in Argentina: the views of public and private sector representatives. Cad Saude Publica [Internet]. 2017 Jul 3;33(6):e00014316. Available from: http://www.ncbi.nlm.nih.gov/pubmed/28678932

24. Knai C, Scott C, D’Souza P, James L, Mehrotra A, Petticrew M, et al. The Public Health Responsibility Deal: making the workplace healthier? J Public Health (Oxf) [Internet]. 2017 Jun 1;39(2):373–86. Available from: http://www.ncbi.nlm.nih.gov/pubmed/27302202

25. Durand MA, Petticrew M, Goulding L, Eastmure E, Knai C, Mays N. An evaluation of the Public Health Responsibility Deal: Informants’ experiences and views of the development, implementation and achievements of a pledge-based, public-private partnership to improve population health in England. Health Policy [Internet]. 2015 Nov;119(11):1506–14. Available from: http://www.ncbi.nlm.nih.gov/pubmed/26433565

26. Knai C, Petticrew M, Durand MA, Eastmure E, Mays N. Are the Public Health Responsibility Deal alcohol pledges likely to improve public health? An evidence synthesis. Addiction [Internet]. 2015 Aug;110(8):1232–46. Available from: http://www.ncbi.nlm.nih.gov/pubmed/25807862

27. Knai C, Petticrew M, Durand MA, Scott C, James L, Mehrotra A, et al. The Public Health Responsibility deal: Has a public-private partnership brought about action on alcohol reduction? Addiction. 2015;110(8):1217–25.

28. Petticrew M, Douglas N, Knai C, Durand MA, Eastmure E, Mays N. Health information on alcoholic beverage containers: has the alcohol industry’s pledge in England to improve labelling been met? Addiction [Internet]. 2016 Jan;111(1):51–5. Available from: http://www.ncbi.nlm.nih.gov/pubmed/26467551

29. Knai C, Petticrew M, Durand MA, Eastmure E, James L, Mehrotra A, et al. Has a public-private partnership resulted in action on healthier diets in England? An analysis of the Public Health Responsibility Deal food pledges. Food Policy [Internet]. 2015;54(March 2011):1–10. Available from: http://dx.doi.org/10.1016/j.foodpol.2015.04.002

30. Panjwani C, Caraher M. The Public Health Responsibility Deal: brokering a deal for public health, but on whose terms? Health Policy [Internet]. 2014 Feb;114(2–3):163–73. Available from: http://www.ncbi.nlm.nih.gov/pubmed/24309298

31. Knai C, James L, Petticrew M, Eastmure E, Durand MA, Mays N. An evaluation of a public-private partnership to reduce artificial trans fatty acids in England, 2011-16. Eur J Public Health [Internet]. 2017;27(4):605–8. Available from: http://www.ncbi.nlm.nih.gov/pubmed/28339665

32. Knai C, Petticrew M, Scott C, Durand MA, Eastmure E, James L, et al. Getting England to be more physically active: are the Public Health Responsibility Deal’s physical activity pledges the answer? Int J Behav Nutr Phys Act [Internet]. 2015;12:107. Available from: http://www.ncbi.nlm.nih.gov/pubmed/26384783

33. Willis CD, Corrigan C, Stockton L, Greene JK, Riley BL. Exploring the unanticipated effects of multi-sectoral partnerships in chronic disease prevention. Health Policy [Internet]. 2017 Feb;121(2):158–68. Available from: http://www.ncbi.nlm.nih.gov/pubmed/27938850

34. Pérez-Escamilla R. Innovative Healthy Lifestyles School-Based Public-Private Partnerships Designed to Curb the Childhood Obesity Epidemic Globally: Lessons Learned From the Mondelēz International Foundation. Food Nutr Bull [Internet]. 2018 Jun;39(1_suppl):S3–21. Available from: http://www.ncbi.nlm.nih.gov/pubmed/29756491

35. Schoeppe J, Cheadle A, Melton M, Faubion T, Miller C, Matthys J, et al. The Immunity Community: A Community Engagement Strategy for Reducing Vaccine Hesitancy. Health Promot Pract [Internet]. 2017;18(5):654–61. Available from: http://www.ncbi.nlm.nih.gov/pubmed/28398837

36. Garcia-Silva B, Handler E, Wolfe J. A Public-Private Partnership to Mitigate Food Insecurity and Food Waste in Orange County, California. Am J Public Health [Internet]. 2017 Jan;107(1):105. Available from: http://www.ncbi.nlm.nih.gov/pubmed/27736209
