# Supplementary material for: Promoting population health with public-private partnerships: Where’s the evidence?
Source: BMC Public Health. 2019 Nov 1;19:1438. doi: 10.1186/s12889-019-7765-2 (PMC6824113; doi:10.1186/s12889-019-7765-2)
Supplement: Supplementary file 3 — Additional file 3: Table S1. Details and classification of the 25 PPPs evaluated. [file 12889_2019_7765_MOESM3_ESM.docx]

**Table S1:** Characteristics and classification of the 25 public-private partnerships evaluated

| **Name of PPP Evaluated** (reference S2 appendix) | **Health problem** | **Independence** | **Competing interests private partner** | **Country** | **Methodology of evaluation** | **Evaluation Type** | **View regarding PPPs** |
| --- | --- | --- | --- | --- | --- | --- | --- |
| Fresh to You program (1) | NCDs | NO | low potential | USA | Mixed-methods | Process with quantitative intermediate outcome variables | Supportive |
| Healthy Weight Commitment Foundation (2,3) | NCDs | UNCLEAR | high potential | USA | Quantitative | Process with quantitative intermediate outcome variables | Semi-critical |
| HOME GROWN (HG)Food activistm and Urban Agricuture in Milwaukee, Wisconsin. (4) | NCDs | YES | low potential | USA | Qualitative | Process without quantitative intermediate outcome variables | Semi-critical |
| KINET (5) | Infectious Disease (TB, HIV, Malaria) | NO | low potential | Tanzania | Mixed-methods | Process with quantitative intermediate outcome variables | Supportive |
| Let’s Move salad bars to schools (LMsb2s) (6) | NCDs | NO | low potential | USA | Quantitative | Process with quantitative intermediate outcome variables | Supportive |
| Million Hearts (7) | NCDs | NO | low potential | USA | Qualitative | Process without quantitative intermediate outcome variables | Supportive |
| National Malaria Control Programme (NMCP). (8) | Infectious Disease (TB, HIV, Malaria) | NO | low potential | Zambia | Quantitative | Impact with health related outcomes | Supportive |
| No specific name (is a partnership between Malaysian Malaria Control Programme MCP and a private palm oil, rubber and acacia plantation in the state of Sabah). (9) | Infectious Disease (TB, HIV, Malaria) | NO | some potential | Malaysia | Qualitative | Process without quantitative intermediate outcome variables | Supportive |
| No specific name: Effective Health Care Alliance Research Programme (EHCARP-Nigeria), in collaboration with Nigeria National Petroleum Cooperation and Mobil Producing Nigeria Unlimited (NNPC/MPN) Joint Venture (10) | Infectious Disease (TB, HIV, Malaria) | NO | low potential | Nigeria | Quantitative | Impact with health related outcomes | Supportive |
| No specific name: It is a partnership of the Food Standards Agency (FSA)with the food industry to encourage product reformulation as de second part ot its salt intake reduction campaign. (11) | NCDs | YES | high potential | UK | Quantitative | Process with quantitative intermediate outcome variables | Supportive |
| No specific name: TEDHA as a partner to the National Anti- Malaria Campaign (12) | Infectious Disease (TB, HIV, Malaria) | NO | low potential | Sri Lanka | Quantitative | Process with quantitative intermediate outcome variables | Supportive |
| Parklives (13) | NCDs | YES | high potential | UK | Qualitative | Process without quantitative intermediate outcome variables | Critical |
| Partnership for a Healthier America (PHA) (First Lady Michelle Obama’s Let’s Move initiative) (14) | NCDs | YES | high potential | USA | Qualitative | Process without quantitative intermediate outcome variables | Critical |
| Tanzanian National Voucher Scheme (TNVS) (15,16) | Infectious Disease (TB, HIV, Malaria) | NO | Low potential | Tanzania | Quantitative | Process with quantitative intermediate outcome variables | supportive |
| The African Comprehensive HIV/AIDS Partnerships (ACHAP) (17) | Infectious Disease (TB, HIV, Malaria) | NO | low potential | Botswana | Qualitative | Process without quantitative intermediate outcome variables | Tentatively supportive |
| The Australian Food and Health Dialogue / Healthy Food Partnership (18–22) | NCDs | UNCLEAR | high potential | NA | Mixed-methods | Process with quantitative intermediate outcome variables | Critical |
| The Less Salt, More Life program (23) | NCDs | YES | high potential | Argentina | Qualitative | Process without quantitative intermediate outcome variables | Supportive |
| The Public Health Responsibility Deal – Health at work (24,25) | Other | YES | some potential | UK | Qualitative | Process without quantitative intermediate outcome variables | Critical |
| The Public Health Responsibility Deal - Alcohol Pledge (25–28) | NCDs | YES | high potential | NA | Mixed-methods | Process with quantitative intermediate outcome variables | Critical |
| The Public Health Responsibility Deal - Food Pledges (25,29–31) | NCDs | YES | high potential | NA | Qualitative | Process without quantitative intermediate outcome variables | Critical |
| The Public Health Responsibility Deal - Physical Activity Pledge (25,32) | NCDs | YES | high potential | UK | Qualitative | Process without quantitative intermediate outcome variables | Critical |
| Various: Multi-sectoral Partnerships to Promote Healthy Living and Prevent Chronic Disease (MSP) (33) | NCDs | NO | some potential | Canada | Qualitative | Process without quantitative intermediate outcome variables | Tentatively supportive |
| Various: There are several PPPs in 7 different countries always with the same private partner the Mondeléz International Foundation (MIF) (34) | NCDs | NO | high potential | Various in Asia Europe  and Latin America. | Qualitative | Process without quantitative intermediate outcome variables | Supportive |
| Vax Northwest (35) | Other | NO | low potential | USA | Mixed-methods | Process with quantitative intermediate outcome variables | Supportive |
| “Waste Not Orange County.” (36) | Other (urban food insecurity) | NO | some potential | USA | Qualitative | Process with quantitative intermediate outcome variables | Supportive |
