# Supplementary material for: Promoting population health with public-private partnerships: Where’s the evidence?
Source: BMC Public Health. 2019 Nov 1;19:1438. doi: 10.1186/s12889-019-7765-2 (PMC6824113; doi:10.1186/s12889-019-7765-2)
Supplement: Supplementary file 4 — Additional file 4: Table S2. Analysis of quality of the 36 studies evaluating PPPs included in the review. [file 12889_2019_7765_MOESM4_ESM.docx]

**Table S2:** Quality evaluation of the 36 evaluations of public-private partnerships

| **First, Author Year [reference in text S2]** | **1 INDEPENDENCE** | **2 OBJECTIVES** | **3A PROCEDURES DATA COLLECTION** | **3B CONFOUNDING** | **4A QUANTI OUTCOME MEASUREMENT** | **4B QUALI OUTCOME MEASUREMENT** | **GLOBAL RATING** |
| --- | --- | --- | --- | --- | --- | --- | --- |
| Asuquo, 2015 [10] | HIGH | LOW | HIGH | HIGH | HIGH | N/A | WEAK |
| Castronuovo, 2018 [23] | LOW | LOW | N/A | N/A | N/A | LOW | STRONG |
| Durand, 2015 [25] | LOW | LOW | N/A | LOW | N/A | LOW | STRONG |
| Elliott, 2014 [18] | MODERATE | MODERATE | N/A | MODERATE | N/A | LOW | MODERATE |
| Eze, 2014 [15] | HIGH | LOW | LOW | MODERATE | LOW | N/A | MODERATE |
| Fernando, 2018 [12] | HIGH | MODERATE | HIGH | HIGH | LOW | N/A | WEAK |
| Garcia-Silva, 2017 [36] | HIGH | MODERATE | HIGH | MODERATE | HIGH | N/A | WEAK |
| Gorham, 2015 [1] | HIGH | LOW | LOW | MODERATE | HIGH | MODERATE | WEAK |
| Harris, 2012 [6] | HIGH | HIGH | HIGH | MODERATE | MODERATE | N/A | WEAK |
| Ramiah, 2006 [17] | HIGH | MODERATE | N/A | N/A | N/A | MODERATE | MODERATE |
| Jane, 2018 [13] | LOW | LOW | N/A | LOW | N/A | LOW | STRONG |
| Jones, 2016 [21] | MODERATE | LOW | LOW | LOW | N/A | MODERATE | MODERATE |
| Knai, 2017 [31] | LOW | LOW | N/A | LOW | N/A | LOW | STRONG |
| Knai, 2015 [29] | LOW | LOW | N/A | LOW | N/A | LOW | STRONG |
| Knai, 2015 [26] | LOW | LOW | N/A | N/A | N/A | LOW | STRONG |
| Knai, 2015 [32] | LOW | LOW | N/A | LOW | N/A | LOW | STRONG |
| Knai, 2017 [24] | LOW | LOW | N/A | LOW | N/A | LOW | STRONG |
| Knai, 2015 [27] | LOW | LOW | N/A | LOW | N/A | LOW | STRONG |
| Kramer, 2017 [16] | HIGH | LOW | MODERATE | MODERATE | MODERATE | MODERATE | MODERATE |
| Leon, 2017 [14] | LOW | MODERATE | N/A | N/A | N/A | MODERATE | MODERATE |
| Lindberg, 2017 [19] | LOW | LOW | N/A | LOW | N/A | LOW | STRONG |
| Ng, 2014 [2] | MODERATE | LOW | LOW | LOW | LOW | NA | STRONG |
| Ng, 2014 [3] | MODERATE | LOW | LOW | LOW | LOW | NA | STRONG |
| Panjwani, 2014 [30] | LOW | MODERATE | N/A | LOW | N/A | LOW | STRONG |
| Pérez-Escamilla, 2018 [34] | HIGH | LOW | N/A | N/A | N/A | HIGH | WEAK |
| Petticrew, 2016 [28] | LOW | LOW | LOW | LOW | LOW | N/A | STRONG |
| Pettygrove, 2018 [4] | LOW | LOW | N/A | LOW | NA | MODERATE | STRONG |
| Sanders, 2014 [9] | HIGH | MODERATE | N/A | N/A | N/A | MODERATE | MODERATE |
| Schellenberg ,1999 [5] | HIGH | LOW | MODERATE | LOW | LOW | NA | MODERATE |
| Schoeppe, 2017 [35] | HIGH | LOW | LOW | MODERATE | HIGH | MODERATE | WEAK |
| Sedlmayr, 2013 [8] | HIGH | LOW | LOW | LOW | LOW | N/A | MODERATE |
| Shankar, 2013 [11] | LOW | LOW | LOW | LOW | LOW | NA | STRONG |
| Trevena 2014 [20] | LOW | LOW | LOW | LOW | LOW | N/A | STRONG |
| Trevena 2014 [22] | MODERATE | LOW | LOW | MODERATE | LOW | NA | MODERATE |
| Varda DM 2018 [7] | HIGH | LOW | N/A | N/A | MODERATE | N/A | MODERATE |
| Willis 2017 [33] | HIGH | LOW | N/A | N/A | N/A | LOW | MODERATE |
