# Supplementary material for: Promoting population health with public-private partnerships: Where’s the evidence?
Source: BMC Public Health. 2019 Nov 1;19:1438. doi: 10.1186/s12889-019-7765-2 (PMC6824113; doi:10.1186/s12889-019-7765-2)
Supplement: Supplementary file 5 — Additional file 5: Table S3. Conclusions regarding the public-private partnerships (PPP) for health promotion according to characteristics of the PPP or the evaluation, stratified by health problem. [file 12889_2019_7765_MOESM5_ESM.docx]

**Table S3:** Conclusions regarding the public-private partnerships (PPP) for health promotion according to characteristics of the PPP or the evaluation, stratified by health problem

|  | **Non-communicable Disease** | | | | **Infectious disease (TB, Malaria, HIV)** | | | |
| --- | --- | --- | --- | --- | --- | --- | --- | --- |
| **Characteristics of the PPP or the evaluation** |  | Conclusions regarding PPP in health promotion | | |  | Conclusions regarding PPP in health promotion | | |
|  | **N total** | **Critical / Semi-critical**  **N (%)** | **Supportive / Tentatively supportive**  **N (%)** | **P Value** | **N total** | **Critical / Semi-critical**  **N (%)** | **Supportive / Tentatively supportive**  **N (%)** | **P Value** |
| **Potential for conflict between business interests of private partner and the health promotion activity** |  |  |  | 0.152 |  |  |  | NA |
| High potential | 10 | 7 (70.0) | 3 (30.0) |  | 0 | 0 (0.0) | 0 (0.0) |  |
| Moderate potential | 1 | 0 (0.0) | 1 (100.0) |  | 1 | 0 (0.0) | 1 (100.0) |  |
| Low potential | 4 | 1 (25.0) | 3 (75.0) |  | 6 | 0 (0.0) | 6 (100.0) |  |
| **Independence of evaluation** |  |  |  | 0.013 |  |  |  | NA |
| Yes | 9 | 7 (77.8) | 2 (22.2) |  | 0 | 0 (0.0) | 0 (0.0) |  |
| No | 1 | 1 (100.0) | 0 (0.0) |  | 7 | 0 (0.0) | 7 (100.0) |  |
| Unclear | 5 | 0 (0.0) | 5 (100.0) |  | 0 | 0 (0.0) | 0 (0.0) |  |
| **Quality of evaluation** |  |  |  | 0.125 |  |  |  | NA |
| Strong | 8 | 6 (75.0) | 2 (25.0) |  | 0 | 0 (0.0) | 0 (0.0) |  |
| Moderate | 4 | 2 (50.0) | 2 (50.0) |  | 5 | 0 (0.0) | 5 (100.0) |  |
| Weak | 3 | 0 (0.0) | 3 (100.0) |  | 2 | 0 (0.0) | 2 (100.0) |  |
| **Total** | 15 | 8 (53.3) | 7 (46.7) |  | 7 | 0 (0.0) | 7 (100.0) |  |

*Footnote: Evaluations of 25 PPPs from 36 academic studies.*
